# Flow-controlled ventilation decreases mechanical power in postoperative ICU patients

**DOI:** 10.1186/s40635-024-00616-9

**Published:** 2024-03-19

**Authors:** Julien P. Van Oosten, Juliette E. Francovich, Peter Somhorst, Philip van der Zee, Henrik Endeman, Diederik A. M. P. J. Gommers, Annemijn H. Jonkman

**Affiliations:** 1 Intensive Care Volwassenen, Erasmus Medical Center, Dr. Molewaterplein 40, 3015 GD Rotterdam, The Netherlands; 2 Technical Medicine Program, Delft University of Technology, Delft, The Netherlands

**Keywords:** Flow-controlled ventilation, FCV, ICU, Electrical impedance tomography, Mechanical power

## Abstract

**Background:**

Mechanical power (MP) is the energy delivered by the ventilator to the respiratory system and combines factors related to the development of ventilator-induced lung injury (VILI). Flow-controlled ventilation (FCV) is a new ventilation mode using a constant low flow during both inspiration and expiration, which is hypothesized to lower the MP and to improve ventilation homogeneity. Data demonstrating these effects are scarce, since previous studies comparing FCV with conventional controlled ventilation modes in ICU patients suffer from important methodological concerns.

**Objectives:**

This study aims to assess the difference in MP between FCV and pressure-controlled ventilation (PCV). Secondary aims were to explore the effect of FCV in terms of minute volume, ventilation distribution and homogeneity, and gas exchange.

**Methods:**

This is a physiological study in post-cardiothoracic surgery patients requiring mechanical ventilation in the ICU. During PCV at baseline and 90 min of FCV, intratracheal pressure, airway flow and electrical impedance tomography (EIT) were measured continuously, and hemodynamics and venous and arterial blood gases were obtained repeatedly. Pressure–volume loops were constructed for the calculation of the MP.

**Results:**

In 10 patients, optimized FCV versus PCV resulted in a lower MP (7.7 vs. 11.0 J/min; *p* = 0.004). Although FCV did not increase overall ventilation homogeneity, it did lead to an improved ventilation of the dependent lung regions. A stable gas exchange at lower minute volumes was obtained.

**Conclusions:**

FCV resulted in a lower MP and improved ventilation of the dependent lung regions in post-cardiothoracic surgery patients on the ICU.

*Trial registration* Clinicaltrials.gov identifier: NCT05644418. Registered 1 December 2022, retrospectively registered.

**Supplementary Information:**

The online version contains supplementary material available at 10.1186/s40635-024-00616-9.

## Background

In mechanically ventilated patients with the acute respiratory distress syndrome (ARDS), the development of secondary lung injury and inflammation—also known as ventilator-induced lung injury (VILI), is a major contributor to mortality in the intensive care unit (ICU). Variables associated with the development of VILI have been unified in the mechanical power (MP), a parameter that provides an estimate of the resistive, static and dynamic elastic forces that are transferred from the ventilator to the respiratory system [1]. MP can in part be modulated by ventilator strategy and settings [2]. In conventional controlled mechanical ventilation modes, the inspiratory phase is controlled and expiration is a passive process. The energy that is dissipated to the lung parenchyma during the expiratory phase has largely been ignored in the assessment of VILI risks. However, a sudden decrease in airway pressure may also contribute to VILI development considering alveolar heterogeneity and the potential for alveolar collapse and atelectrauma [3, 4].

Flow-controlled ventilation (FCV) is a new ventilation mode that uses a constant low flow during both inspiration and expiration, resulting in a linear increase in airway pressure during inspiration and a linear decrease in airway pressure during expiration [5]. The controlled expiration is accomplished by forcing the continuous flow through a nozzle in the ventilator, thereby generating a negative pressure in the tubing system. Through this Bernoulli effect, the ventilator controls the expiratory flow rate and thereby generates the linear decrease in airway pressure [5]. Theoretically, this could lower the energy dissipation during expiration, and hence lower the MP as compared to volume- or pressure-controlled ventilation (VCV and PCV, respectively) [5, 6]. Furthermore, FCV reduced alveolar heterogeneity and improved lung aeration on CT scan in a porcine model of ARDS [7], which also resulted in increased ventilation efficiency (lower minute volume with stable PaCO_2_) and attenuated lung injury. By lowering the MP and providing more homogeneous ventilation, FCV could thus be especially beneficial in critically ill patients requiring prolonged mechanical ventilation in the ICU.

To date, only two small pilot studies examined the physiological effects of FCV in the ICU, where a lower MP [8] and better oxygenation [9] with FCV were reported. However, results cannot be interpreted reliably since in both studies airway pressures were measured at different levels (at the circuit valve in volume-targeted ventilation versus intratracheally with FCV), thereby directly affecting MP calculations. The effects of FCV on lung homogeneity in ventilated ICU patients have not yet been investigated.

To better understand the concept and potential beneficial effects of FCV, we designed a physiological study comparing FCV and PCV in postoperative cardiothoracic surgery patients with relatively healthy lungs requiring mechanical ventilation at the ICU. The primary aim of this report was to assess the differences in MP, hypothesizing a lower MP with FCV. Secondary aims were to explore the effect of FCV in terms of minute volume, dissipated energy, ventilation distribution and homogeneity, and gas exchange.

## Methods

For additional details, see online additional data, which is accessible from this issue’s table of content online at https://www.icm-experimental.springeropen.com.

### Study design and patients

This prospective interventional study was conducted at the ICU of the Erasmus Medical Center, Rotterdam, The Netherlands, from February 2022 to May 2023 (ClinicalTrials.gov: NCT05644418), after approval by the local Medical Ethics Committee. Adult cardiothoracic surgery patients requiring postoperative controlled mechanical ventilation in the ICU were screened and provided written informed consent prior to their surgery. Eligibility was reassessed at ICU arrival. Enrollment criteria were (1) invasive controlled mechanical ventilation, (2) FiO_2_ ≤ 50%, (3) positive end-expiratory pressure (PEEP) ≤ 10 cmH_2_O. Exclusion criteria were: (1) severe sputum stasis, (2) severe respiratory insufficiency (i.e., PaO_2_/FiO_2_ < 100 mmHg, or moderate-to-severe ARDS according to the Berlin definition [10]), (3) untreated pneumothorax, (4) hemodynamic instability, (5) contraindications to EIT monitoring and (6) an inner tube diameter ≤ 6 mm.

### Data collection

At enrollment, we collected sex, age, body mass index (BMI), ideal body weight (IBW), medical history, type of surgery, and hemodynamic status [noradrenalin dose, central venous oxygen saturation (ScvO_2_) and arterial-venous CO_2_ gap].

### Continuous monitoring

A conventional tube adapter (Ventinova Medical BV, The Netherlands) and flow sensor (Hamilton Medical, Switzerland) were placed in between the endotracheal tube and ventilator tubing. This tube adapter is an essential part of FCV (Evone ventilator, Ventinova Medical BV) and consists of a thin pressure probe with a length of approximately 25 cm from the endotracheal tube opening, hence allowing the measurement of intratracheal pressures. Output of this pressure probe and the flow sensor were connected to a dedicated signal acquisition system (MP160, BIOPAC Systems Inc., USA) for a synchronized recording of waveforms sampled at 200 Hz (AcqKnowledge, BIOPAC Systems Inc., USA) during the full protocol. To assess homogeneity of ventilation, continuous monitoring with electrical impedance tomography (EIT) was initiated with a belt placed at the 4th–5th intercostal space (PulmoVista 500, Dräger Medical, Germany).

### Study procedures

**Fig. 1:**
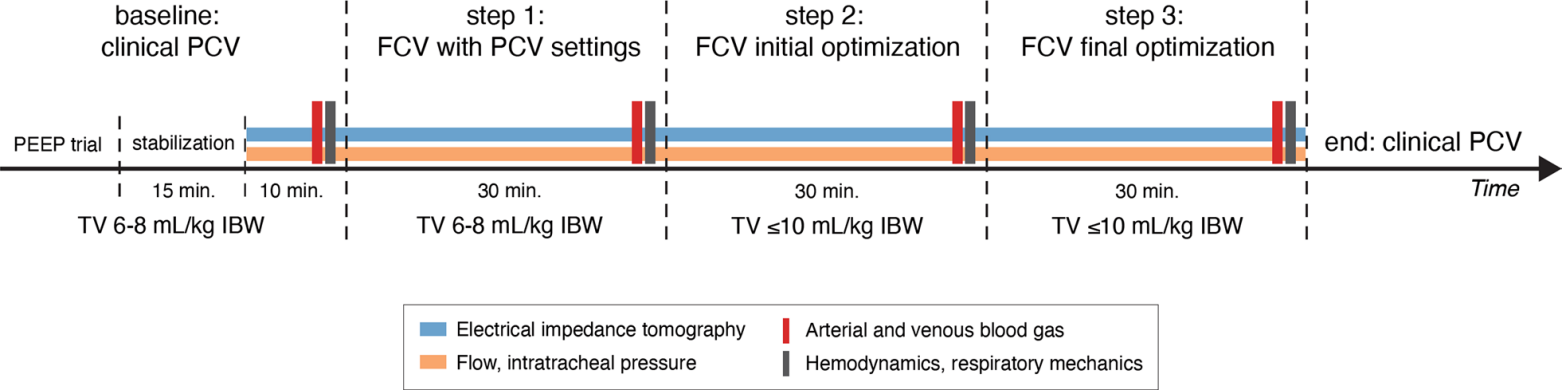
Study procedures with corresponding measurements

The study protocol was initiated directly after surgery when the patient arrived on the ICU. No recruitment maneuvers were performed after weaning from the cardiopulmonary bypass. Study steps are presented in Fig. 1 and lasted 30 min each. Arterial blood gases (ABG), central venous blood gases, SpO_2_, and hemodynamic and respiratory mechanics measurements were obtained at the end of each step. We aimed for a SpO_2_ of 95–100%, PaO_2_ < 15 kPa and end-tidal CO_2_ (EtCO_2_) and PaCO_2_ between 4.5 and 6.5 kPa throughout the protocol.

- *Baseline: PCV.* Settings were optimized according to our local protocol (with PEEP as per a decremental PEEP trial aiming for the highest dynamic compliance (maximum PEEP 24 cmH_2_O), and tidal volumes of 6–8 mL/kg IBW) and were kept for at least 15 min to reach a stable condition before initiating continuous EIT, pressure and flow recordings for another 10 min at this step.
- *Step 1: FCV at ‘similar’ PCV settings.* To directly compare FCV and PCV, the ventilation mode was switched to FCV with the same PEEP and FiO_2_ settings as baseline. Ppeak was titrated to reach the same tidal volumes as with PCV. Continuous set flow (determining the minute ventilation) was titrated to maintain a stable EtCO_2_. Inherent to the FCV working mechanism with an I:E ratio of 1:1, respiratory rate is the direct result of the combination of the set flow, the pressure difference between PEEP and Ppeak and the patient’s respiratory mechanics (resistance and compliance). Settings were kept for 30 min.
- *Step 2: FCV initial optimization.* To maximally utilize the FCV working mechanism, FCV was optimized according to the ABG at the end of step 1 and the manufacturer’s instructions. PEEP was kept constant and FiO_2_ was adapted, if necessary, based on the PaO_2_ and target values mentioned at baseline. Ppeak was titrated in steps of 1 cmH_2_O to reach the highest dynamic compliance: if tidal volume increased more than expected (based on the dynamic compliance) when increasing the Ppeak, then Ppeak was further increased with 1 cmH_2_O. This sequence was repeated until tidal volumes did not increase more than expected (thus decreasing dynamic compliance) or until a safety limit of 10 mL/kg IBW was reached. Flow was adjusted to maintain PaCO_2_ within target values. Settings were kept for 30 min.
- *Step 3: FCV final optimization. *Based on the ABG of step 2, flow and FiO_2_ were adjusted, if necessary, to maintain PaO_2_ and PaCO_2_ within target values.

The patient’s management was then resumed as per local clinical protocol (with PCV settings similar as baseline).

### Offline analysis

Computation of parameters was performed for the steps baseline, step 1 and step 3.

#### Flow and pressure tracings

Breath-by-breath analysis of flow and intratracheal pressure was performed (Matlab 2021a, MathWorks, USA) for a period of 8–10 min at the end of each step. From the flow tracings, inspiratory time (Ti), RR, tidal volume (time-integral of inspiratory flow) and minute volume were calculated. Ppeak, total PEEP, and mean pressure were derived from the pressure waveforms. From the constructed pressure–volume (PV) loops (Additional file 1: Figure S1), the total energy per breath was computed as the integral of the PV loop times 0.098 (conversion to Joule), including the elastic dynamic and resistive components, but not the static part (unknown PEEP volume). The MP (Joule/min) was calculated by multiplying the total energy per breath by the RR. Dissipated energy was computed as the hysteresis area of the PV loop per breath (in Joule/Liter). For comparison, we also calculated the MP using bedside formulas for PCV [11] and using the simplified equation from Gattinoni [12] for FCV (which is similar to VCV during inspiration with its continuous flow).

#### EIT

Pixel-level EIT data were obtained (PV500 Data Analysis SW130) and processed using a custom software developed in Python. At the end of each step, a stable period of at least 10 breaths was manually selected. For each pixel, an average inspiration was computed over this stable period (Additional file 1: Figure S2). Pixels with a tidal impedance change (∆*Z*) of at least 15% of the maximum pixel ∆*Z* were included in the analysis (Additional file 1: Figure S3), assuming significant contribution to the ventilated lung space and to minimize influence of cardiac-related artifacts, in line with [13].

Regions of interest (ROIs; ventral, mid-ventral, mid-dorsal and dorsal) were defined with a physiological approach utilizing the ventilated lung space: per patient, we first computed an average pixel impedance map of all three steps (baseline, step 1 and step 3). Then, ROIs were defined with each ROI representing 25% of the total variation in lung impedance of this average map (Additional file 1: Figures S4, S5). This computation allowed to assess subtle changes in regional EIT parameters between PCV and FCV.

For each step, the global ∆*Z* and regional ∆*Z* (per ROI) were calculated, as well as the global and regional static compliance (per ROI) (i.e., ∆*Z*/driving pressure, with driving pressure being the difference between plateau pressure and total PEEP derived from the intratracheal pressure tracings during both PCV and FCV), and the change in global end-expiratory lung impedance (∆EELI). Furthermore, we visualized and quantified the overall, spatial and temporal ventilation homogeneity as follows:

- Global inhomogeneity index (GI) [14]; GI(%) = ((∑[*x*,*y* ∈ _lung_]|∆*Z*_*xy*_ – Median(∆*Z*_lung_)|)/(∑[*x*, ∈ _lung_]∆*Z*_*xy*_)) × 100%; with ∆*Z*_*xy*_ the impedance change of a ventilated pixel (*x*,*y*) and ∆*Z*_lung_ the impedance change of the ventilated lung space.
- Regional spatial volume distribution: first, to provide a visualization of the continuous regional inspiratory volume distribution, impedance waveforms per ROI were normalized over time and visualized as a percentage of the global ∆*Z* (Additional file 1: Figure S6). Second, regional intra-tidal impedance distribution was visualized by dividing the global inspiration into five parts of equal ∆Z and then computing the ∆*Z* for each ROI (Additional file 1: Figure S7) [15].
- Regional ventilation delay index (RVDi) [16]: regional ventilation delay (RVD) was first computed as RVD = Δ*t*_RVD_/Δ*t*_max–min_, with Δ*t*_RVD_ the time between the start of inspiration (as per the global ∆*Z*) until pixel ∆*Z* reached 40% of the maximal ∆*Z*; this was normalized to the global inspiration time (Δ*t*_max–min_) and expressed as percentage (Additional file 1: Figure S8). RVDi was then calculated as the standard deviation of all pixel RVDs; a lower RVDi reflects a more homogeneous temporal lung inflation.

#### Hemodynamics and gas exchange

PaO_2_, PaCO_2_, PaO_2_/FiO_2_ ratio, central venous oxygen saturation (ScvO_2_), arterial-venous CO_2_ gap, ventilatory ratio [17], and noradrenalin dose were obtained per step.

#### Primary endpoint and secondary exploratory endpoints

Initially, our primary endpoint (see Clinicaltrials.gov: NCT05644418) was the EELI difference between PCV and FCV (step 1) to assess direct changes in lung aeration. FCV at step 1 was chosen for comparison because subsequent FCV optimization could influence EELI due to tidal recruitment. Upon reviewing preliminary data halfway during the study, however, it became apparent that EELI changes could not be evaluated reliably due to EELI changes likely related to clinical fluid therapy in this selected cohort; this was confirmed after enrolling another five patients where continuous EIT monitoring for 2 h on PCV was performed (Additional file 1: Figures S9, S10). Since EELI changes can only be reliably assessed when hemodynamics and fluid administration are relatively stable over the period of interest—which was especially challenging in our population of interest, we changed our primary endpoint to the difference in MP between PCV and optimized FCV for a more robust evaluation. Secondary endpoints were the difference in minute volume, dissipated energy, ventilation distribution and homogeneity, and gas exchange between PCV and (optimized) FCV.

### Sample size

Due to the lack of comparator data, our sample size was based on the reported effect of FCV on minute volume in healthy pigs [18]. Using a matched pairs *T*-test approach (alpha, 0.05; power, 0.80) G*Power (Statistical Power Analyses, Universität Düsseldorf, Germany), the sample size was 6 patients. Since the effect size of FCV on other physiological parameters was unknown, we decided to enroll 10 patients in total. After changing the primary endpoint to the mechanical power, we did not re-power the sample size, because a good estimation of the difference in mechanical power between PCV and FCV could not be established using the previous literature, concerning that in those studies airway pressures during PCV and FCV were measured at different levels.

### Statistical analysis

Statistical analysis was performed using SPSS (IBM, Armonk, USA). Values are presented as median (interquartile range) and were tested for normality using the Shapiro–Wilk test. Steps were compared using the repeated measures ANOVA or the related-samples Friedman’s test depending on the distribution, and with Bonferroni correction for multiple comparisons. *p* < 0.05 was considered statistically significant.

## Results

### Population and characteristics

In total, 21 patients provided informed consent prior to their planned surgery for participating in the full study protocol, of which 10 patients finally participated; their main characteristics are presented in Table 1. Reasons for withdrawal of 11 patients were direct postoperative extubation (*n* = 2), technical problem with the FCV ventilator (*n* = 1), a last-minute canceled and rescheduled surgery (to another day without availability of the study team) (*n* = 6), limited study staff (*n* = 1) and a surgical complication with need to return the patient to the operating theatre (*n* = 1). All patients underwent a median sternotomy (no minimally invasive procedures or off-pump procedures were performed). No serious adverse events were reported. One patient was excluded from EIT analysis due to artifacts in the recordings, likely due to a small ventral pneumothorax that was missed at enrollment.

**Table 1:** Main characteristics of the study population

Characteristic	Total (*N* = 10)
Age, years; median (IQR)	66 (62–70)
Male, sex; *n* (%)	7 (70)
BMI, kg/m^2^; median (IQR)	30.5 (26.4–36.0)
IBW, kg; median (IQR)	71.5 (59.7–73.3)
Medical history (*n*)	
COPD	1
Coronary artery disease	6
Aortic valve stenosis	4
Aneurysm thoracic aorta	1
Hypertrophic cardiomyopathy	1
Endocarditis	1
Type of surgery performed (*n*)	
CABG	4
CABG + AVR	1
AVR	2
AVR + MVP	1
Myectomy + MVP	1
Bentall (aortic replacement)	1
Cardiopulmonary bypass time, minutes; median (IQR)	158 (116–203)
Hemodynamic status	
Intraoperative fluid balance, Liters; median (IQR)	+ 2.30 (1.06–3.14)
Fluid administration during study^a^, Liters; median (IQR)	0.50 (0.04–1.13)
Blood loss during study, Liters; median (IQR)	0.10 (0.04–0.11)
Dose noradrenalin at start study, ug/kg/min; median (IQR)	0.11 (0.05–0.16)
ScvO_2_ at start study, %; median (IQR)	71.2 (64.7–75.8)
Arterial-venous CO_2_ gap at start study, kPa; median (IQR)	1.06 (0.86–1.13)

*AVR* aortic valve replacement, *CABG* coronary artery bypass graft, *COPD* chronic obstructive pulmonary disease, *BMI* Body Mass Index, *IBW* Ideal Body Weight, *IQR* Inter Quartile Range, *MVP* mitral valve repair

^a^To note, the large range can be explained by the fact that some patients received cell saver blood transfusion in the ICU during the study (included in the calculation) while others received it the operating theatre (not included in the calculation)

### Switch from PCV to FCV with similar settings (baseline vs. step 1)

FCV with ‘similar’ PCV settings did not affect MP [9.4 (8.0–11.1) vs. 11.0 (8.5–12.8) J/min, *p* = 0.286] but resulted in a lower dissipated energy [0.22 (0.17–0.26) vs. 0.34 (0.21–0.43) J/L, *p* = 0.008] for FCV vs. PCV, respectively. For all results comparing FCV step 1 and PCV, see Additional file 1: Tables S1 and S2. Although the global inhomogeneity index did not change (Additional file 1: Table S2), FCV with similar PCV settings did result in a more homogeneous spatial ventilation distribution with increased participation of the dorsal lung regions (Additional file 1: Figure S11).

### FCV optimization (baseline vs. step 3)

**Fig. 2:**
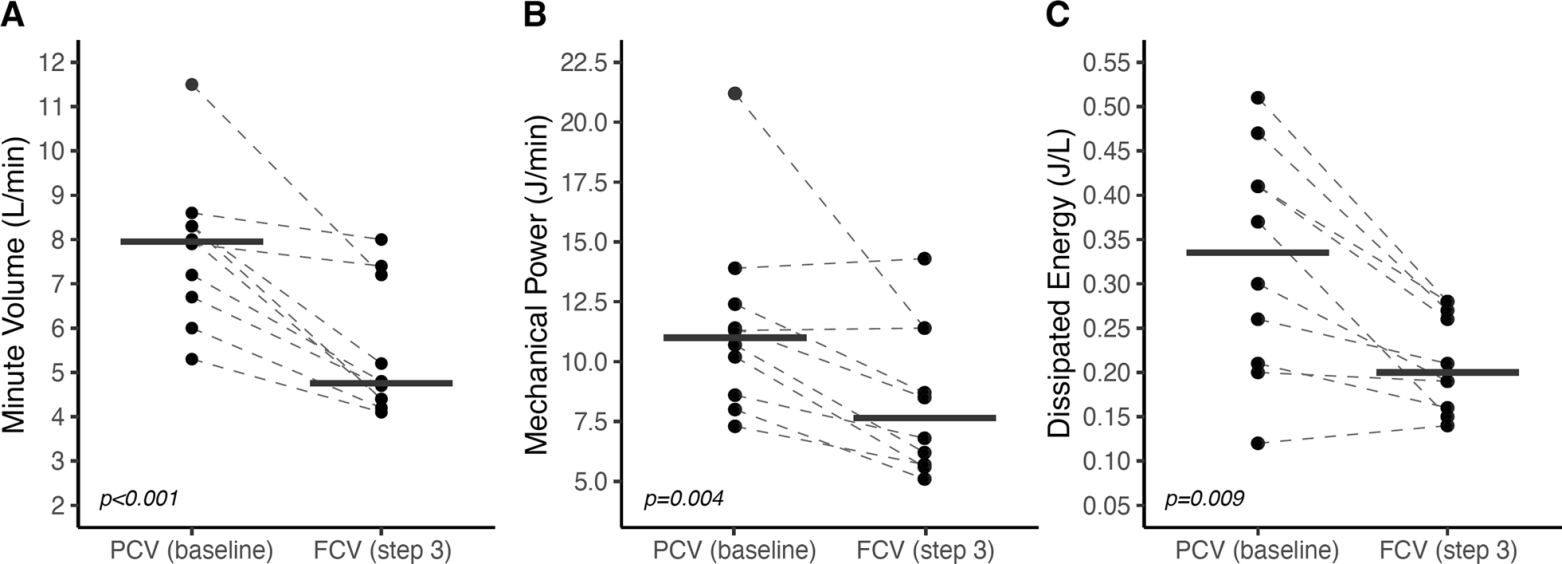
Minute volume, mechanical power and dissipated energy decrease during FCV vs. PCV. Individual data points as well as the group median (horizontal bar) is provided

Since maintaining similar tidal volumes as to conventional controlled ventilation does not utilize the potential of the FCV mode (i.e., tidal recruitment followed by controlled expiration to keep the lungs open), we optimized FCV in the following steps. Optimization of FCV (step 3) resulted in a significantly lower MP and dissipated energy compared to PCV at baseline while the minute volume and ventilatory ratio decreased (Table 2 & Fig. 2). FCV also resulted in a significantly lower RR, lower airway resistance and higher mean airway pressure. Despite changes in ventilation, oxygenation (PaO_2_ and PaO_2_/FiO_2_), PaCO_2_ and hemodynamics remained stable (Table 2). Two patients demonstrated a relatively high static respiratory system compliance in combination with a high airway resistance; their results are additionally presented in Additional file 1: Table S3.

**Table 2:** Results PCV (baseline) vs. optimized FCV (step 3)

	PCV baseline Median (IQR)	FCV step 3 Median (IQR)	p value
Respiratory parameters			
Inspiratory TV/IBW (mL)	6.0 (5.5–7.1)	8.4 (7.9–8.7)	0.004
Driving pressure (cmH_2_O)	9.2 (7.7–11.7)	11.9 (9.8–14.0)	0.031
PEEP set (cmH_2_O)	7.5 (6.4–8.0)	8.0 (5.8–8.0)	1.000
PEEP total (cmH_2_O)	8.3 (7.5–9.2)	8.4 (7.5–10.1)	1.000
Ppeak set (cmH_2_O)	20.0 (18.8–22.0)	20.5 (19.8–24.3)	0.281
Ppeak measured (cmH_2_O)	18.6 (16.8–21.5)	21.1 (20.2–24.9)	0.012
Pplat (cmH_2_O)	17.5 (16.2–20.5)	20.0 (19.0–24.0)	0.011
Pmean (cmH_2_O)	12.6 (11.0–13.4)	14.7 (13.0–16.9)	< 0.001
Crs static (mL/cmH_2_O)	44.5 (36.1–52.7)	47.0 (39.7–51.8)	1.000
Resistance (cmH_2_O/L/s)	13.8 (12.4–14.9)	8.2 (6.8–9.1)	0.002
RR (x/min)	18 (17.5–20.0)	8.5 (7.6–13.1)	< 0.001
Minute volume (L/min)	8.0 (6.5–8.4)	4.8 (4.4–7.3)	0.001
Mechanical power (J/min)	11.0 (8.5–12.8)	7.7 (5.7–11.4)	0.004
Mechanical power, bedside formulas (J/min)	13.2 (10.5–15.2)	8.0 (5.9–11.6)	0.003
Dissipated energy (J/L)	0.34 (0.21–0.43)	0.20 (0.16–0.27)	0.009
Gas exchange parameters			
P/F ratio	324 (241–365)	300 (273–369)	1.000
PaO_2_ (kPa)	14.3 (12.9–17.7)	13.1 (12.0–13.9)	0.212
PaCO_2_ (kPa)	5.4 (5.1–6.2)	5.3 (5.1–5.9)	0.791
Ventilatory ratio	1.20 (1.11–1.31)	0.75 (0.67–1.15)	0.001
Hemodynamic parameters			
Arterial-venous delta CO_2_ (kPa)	1.06 (0.86–1.13)	0.82 (0.77–1.09)	1.000
ScvO_2_ (%)	71.2 (64.7–75.8)	69.1 (64.1–76.0)	1.000
Dose noradrenalin (ug/kg/min)	0.11 (0.05–0.16)	0.13 (0.04–0.16)	1.000

*Crs* Compliance respiratory system, *FCV* flow-controlled ventilation, *IBW* ideal body weight, *IQR* inter quartile range, *PCV* pressure-controlled ventilation, *Ppeak* peak pressure, *PEEP* positive end-expiratory pressure, *Pmean* mean airway pressure, *Pplat* plateau pressure, *PaO*_*2*_ arterial partial oxygen pressure, *PaCO*_*2*_ arterial partial carbon dioxide pressure, *P/F ratio* PaO_2_/FiO_2_ ratio, *RR* Respiratory Rate, *ScvO*_*2*_ central venous oxygen saturation, *TV* tidal volume

**Fig. 3:**
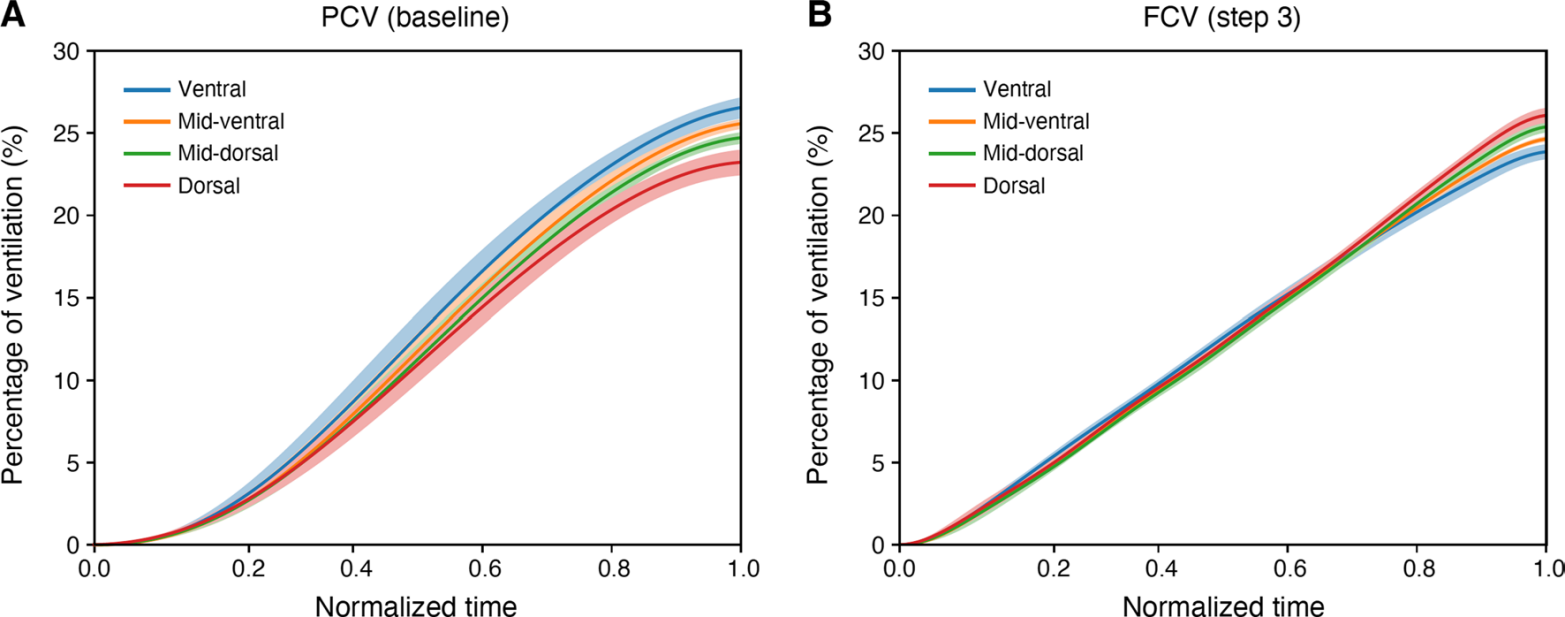
Continuous regional volume distribution: average normalized impedance waveforms with 95% confidence interval per ROI over time and as a percentage of the global ∆*Z*. **A** During PCV (baseline), **B** during optimized FCV (step 3). Compared to PCV at baseline, optimized FCV resulted in a more homogeneous spatial ventilation distribution with increased participation of the dorsal lung regions

EIT results are presented in Table 3. Whereas the dorsal ROI participated less to tidal ventilation during PCV at baseline, there was a significant increase in contribution of the dorsal ROI to tidal ventilation during optimized FCV, even exceeding Δ*Z* values of the ventral ROI (Table 3 and Fig. 3). The higher tidal volumes during optimized FCV did not result in overdistension of the ventral lung as demonstrated by the increase in regional static compliance during optimized FCV compared to PCV for all four ROIs (Table 3). Overall lung homogeneity and temporal ventilation homogeneity, reflected by the GI and RVDi respectively, did not differ between modes. For detailed comparison of ventilation homogeneity parameters of all three study steps, see Additional file 1: Figures S11 and S12.

**Table 3:** EIT results PCV (baseline) vs optimized FCV (step 3); values represent median (IQR)

	Optimized FCV	*p* value
a. Changes in EIT parameters during FCV as compared to PCV^a^		
Global change in ΔZ (%)	59.4 (34.3–72.1)	
Regional change in ΔZ (%)		0.030^1^
ROI ventral	39.7 (22.1–49.5)	
ROI mid-ventral	50.9 (26.6–66.3)	
ROI mid-dorsal	73.6 (34.3–78.6)	
ROI dorsal	81.1 (52.7–104.7)	
Global change in static compliance (%)	13.4 (8.0–26.7)	
Regional change in static compliance (%)		0.017^2^
ROI ventral	2.4 (− 7.0–19.2)	
ROI mid-ventral	15.2 (2.7–24.2)	
ROI mid-dorsal	23.3 (7.1–32.6)	
ROI dorsal	27.5 (19.9–45.2)	
Change in global EELI (a.u.)	53 (− 17–100)	0.163

	PCV	Optimized FCV	*p* value
b. Absolute EIT parameters reflecting lung and ventilation homogeneity			
GI (%)	43.8 (41.4–45.3)	43.5 (39.7–45.7)	1.000
RVDi (%)	2.75 (2.28–4.63)	4.23 (3.39–6.11)	0.717

*a.u.* arbitrary units, *EELI* end-expiratory lung impedance, *EIT* electrical impedance tomography, *FCV* flow-controlled ventilation, *GI* global inhomogeneity index, *PCV* pressure-controlled ventilation, *ROI* region of interest, *RVDi* regional ventilation delay index

^a^Changes in ΔZ and static compliance are expressed as percentage change between FCV step 3 and PCV at baseline, since both are expressed in arbitrary units which makes direct comparisons between patients unreliable

^1^*p* value reflects the significant difference between PCV baseline vs. FCV step 3 regarding the distribution of Δ*Z* among the four ROIs, using a Kruskall Wallis test on the percentage changes from baseline (to account for the fact that Δ*Z* is measured in arbitrary units). ^2^*p* value reflects the significant difference between PCV baseline vs. FCV step 3 regarding the distribution of the change in static compliance among the four ROIs, using a Kruskall Wallis test on the percentage changes from baseline (to account for the fact that Δ*Z* and thereby also the static compliance is measured in arbitrary units)

## Discussion

The main finding of this study comparing flow-controlled mechanical ventilation with PCV in postoperative cardiothoracic patients in the ICU is that optimized FCV provides ventilation with significantly lower MP. As secondary endpoints we found that a stable gas exchange can be achieved with FCV at lower minute volumes and with lower dissipated energy, and that FCV did not provide a better overall lung homogeneity, but resulted in a more homogeneous spatial ventilation distribution with increased participation of the dorsal lung regions.

### FCV to lower mechanical power

MP is independently associated with clinical outcomes, both in patients with and without lung injury [1, 19]. Our results are comparable with the studies of Grassetto and colleagues [8] and Spraider and colleagues [20] who also demonstrated a significantly lower MP on FCV, but important differences should be acknowledged. First, they used pressure and volume data measured before the tube with VCV or PCV, and after the tube during FCV [8, 20]. This makes a comparison between MP calculations unreliable since the energy needed to overcome the tube resistance was not incorporated with FCV. It also explains why the peak pressure was lower with FCV as compared to VCV in the study of Grassetto et al. (23 vs. 27 cmH_2_O for FCV vs. VCV, respectively, *p* < 0.001) [8]. In addition, Grasetto et al. [8] used VCV instead of PCV. We choose to compare FCV with PCV since this is the most commonly used controlled mechanical ventilation mode in the Netherlands. However, since both PCV and VCV modes make use of relatively high flow rates (while FCV flow rate is maximized to 20 L/min) and have a passive expiratory phase, differences in MP compared with FCV are expected to be rather similar for VCV and PCV (at most a small decrease in MP can be achieved with VCV compared to PCV because of the continuous inspiratory flow) [5].

Obtaining all pressure and flow tracings at the same location in the ventilator circuit during both PCV and FCV enabled us to reliably compare respiratory mechanics between both modes. Furthermore, this allowed us to generate detailed PV loops to calculate the MP and the dissipated energy that is considered to contribute to VILI development [5]. This is in contrast with earlier work [8, 20] where only bedside formulas for MP were used and compared. Note that the elastic static component (i.e., PEEP volume) was not integrated in our PV-loop-based calculation due to the unknown PEEP volume, but PEEP levels were similar in both PCV and FCV. We also computed the MP using bedside formulas (that tend to overestimate the true MP [12]) which also confirmed a significantly lower MP on FCV compared to PCV.

### Effects of FCV on lung recruitment and homogeneity

Using a robust EIT analysis of the overall, spatial and temporal homogeneity, we demonstrated that FCV compared to PCV resulted in a more homogeneous spatial ventilation distribution with increased participation of the dorsal lung regions, despite no change in overall ventilation homogeneity and temporal ventilation homogeneity (assessed with the GI and RVDi, respectively).

Previously, in a randomized crossover study, Weber et al. [21] showed that the EELI and mean lung volume decreased less during FCV than during VCV in 23 obese patients undergoing abdominal surgery, indicating improved lung recruitment with FCV. Such trend was not observed in our study. As previously mentioned, it became apparent that EELI changes could not be evaluated reliably in our study, likely due to variations in EELI resulting from clinical fluid management in our selected cohort, as also previously reported [22]. Furthermore, Weber et al. reported that FCV improved regional ventilation distribution [21]. Although the latter conclusion is similar to ours, the data substantiating these conclusions are not. First, their patients only underwent 7 min of ventilation in each mode [21], making it challenging to quantify the amount of lung (de)recruitment owing to FCV and limiting the ability to fully evaluate the effects on regional ventilation. Moreover, they compared two equal-sized ROIs based on 50% of the ventrodorsal diameter [21]. Inherent to such computation, the amount of pixels participating in the ventral and dorsal ventilation could differ between ventilation modes, making it challenging to interpret subtle changes in EIT parameters. Our physiological approach to ROI definition allowed the assessment of more subtle changes in regional EIT parameters between PCV and FCV. Last, Weber [21] presented the decrease in tidal volume per 25% of expiratory impedance change as a parameter to conclude that FCV improves regional ventilation distribution. However, such parameter does not inform about ventilation homogeneity. In fact, they simply demonstrated that the FCV working principle indeed provides a continuous flow during both inspiration and expiration, thereby leading to a linear decrease in tidal volume (impedance) during expiration on FCV. In the study performed by Spraider et al. [20], cardiac surgery patients were either ventilated with FCV or PCV in the operating theatre. A CT scan made directly postoperatively showed a significantly lower amount of non-aerated lung tissue with FCV, which is in line with our findings that FCV leads to increased participation of the dorsal lung regions to ventilation.

### Effects of FCV on ventilation efficiency

The ventilatory ratio is a useful bedside measurement to estimate the amount of dead space and thereby respiratory efficiency during mechanical ventilation [17]. The lower ventilatory ratio during FCV suggests that it could potentially be a parameter of interest when titrating FCV, since the ventilatory ratio is expected to increase when higher tidal volumes result in lung overdistension. Furthermore, a ventilatory ratio < 1 during FCV in our population with relatively healthy lungs suggest that ventilation was more efficient than predicted and/or the CO_2_ production was lower than predicted.

### FCV optimization method

We optimized tidal volumes during FCV based on the best dynamic compliance after stepwise increasing the Ppeak, while not exceeding tidal volumes of 10 mL/kg IBW for safety reasons. This is different from the approach of Grassetto et al. [8] who kept tidal volumes constant and low, and also different from Van Dessel et al. [9] where FCV was applied with even lower tidal volumes and the same respiratory rate as with VCV. Our optimization method maximizes the FCV concept for multiple reasons. First, an improvement in dynamic compliance may indicate recruitment of lung regions, while a decrease in dynamic compliance would indicate that overdistension prevails. We showed that during optimized FCV the compliance as assessed by EIT increased in all lung regions, which is in line with the study by Spraider et al. who used a similar optimization method and CT assessment [20]. Inherent to the FCV working mechanism with an I:E ratio of 1:1, respiratory rate is the direct result of the combination between the set flow, the pressure difference between Ppeak and PEEP, and the patient’s respiratory mechanics (mainly resistance and compliance). Hence, by increasing tidal volumes, the respiratory rate will further decrease, thereby lowering the MP. In addition, with this lower respiratory rate the lung units with a longer time constant have sufficient time for lung inflation, further supporting recruitment [5].

### Strengths and limitations

To date, this is the first physiological study in an ICU population that evaluates the differences in MP, dissipated energy, and detailed ventilation distribution between FCV and PCV with the use of intratracheal pressure and flow sensors and EIT. This overcomes methodological limitations of previous work and creates new evidence for FCV as a novel ventilation mode. However, our study does have some limitations that should be mentioned. First, upon reviewing preliminary data during the study, we changed our primary endpoint from the difference in end-expiratory lung impedance (EELI) to a more robust measure of the MP as derived from PV loops. Of note, the changes in EELI that were likely the result of fluid management in our specific population did not affect the computation of other EIT parameters. Second, we did not randomize between a ventilation sequence, which could have resulted in order effects and the influence of slow recruitment of partly collapsed lungs postoperatively; this may affect results in favor of FCV (more efficient gas exchange). However, by performing a decremental PEEP trial before the start of the study we expect that fast recruitment took place before measurements started. We explored the significance of this potential order effect by analyzing the minute volume and EtCO_2_ of the 5 patients that were additionally enrolled for undergoing EIT measurements during PCV only, and found that minute volume decreased by only 7% in those patients after 90 min of PCV (from 7.7 to 7.2 L/min), with stable ETCO_2_ values. This is in large contrast with the 40% reduction in minute volume that we found with optimized FCV. Third, by increasing tidal volumes during FCV optimization the ventilatory efficiency increased (i.e., the ratio of ventilatory dead space to tidal volume decreased); this could partly explain the decrease in MP in our study. Indeed, Haudebourg et al. [23] found a reduction in MP of 7% when ventilating patients with 7.7 mL/kg IBW (using a low driving pressure strategy that required an increase in tidal volume), as compared to using low tidal volume ventilation (6.1 mL/kg IBW), both in PCV mode. In contrast, the MP decreased with 30% during optimized FCV in our study. Although our tidal volumes during optimized FCV were slightly higher (8.4 mL/kg IBW), there is likely an additional mechanistic effect of the FCV mode on decreasing the MP (i.e., not explained by the increase in tidal volume alone).

### Clinical implications

Our study was performed in postoperative ICU patients with relatively healthy lungs, with degrees of atelectasis primarily influenced by the surgical procedure and cardiopulmonary bypass time instead of lung disease. We choose this population as we first wanted to systematically evaluate the concept and physiological consequences of the FCV mode prior to moving toward ICU patients with hyperinflammatory and heterogeneous lungs. The role of FCV in such population and within a lung-protective ventilation strategy in ARDS is yet unknown and of further ongoing study (see Clinicaltrials.gov: NCT06051188). A point of debate is the acceptation of higher tidal volumes (and driving pressures) in an ARDS population, considering the current guidelines with tidal volumes limited to 4–8 mL/kg IBW [24]. However, ARDS guidelines are based upon research in an era where FCV was not clinically available. With our work we would rather stimulate discussion and potentially a mindset change to the general approach to mechanical ventilation: not primarily focusing on tidal volumes and driving pressures, but increasingly considering viscoelastic properties of the lung tissue, the time it takes to achieve a certain tidal volume, and the potential benefit of additionally controlling the expiratory phase.

In conclusion, optimized FCV as compared to PCV in postoperative cardiothoracic surgery ICU patients resulted in a significantly lower MP and dissipated energy, as well as in a more homogeneous spatial ventilation distribution with increased participation of the dorsal lung regions. The current study provides a good rationale for assessing the role of FCV in ARDS patients where high MP and alveolar heterogeneity resulting in VILI are still major contributors to ICU morbidity and mortality.

### Supplementary Information

**Additional file 1.** Supplementary methods and results.

## Data Availability

The datasets used and/or analyzed during the current study are available from the corresponding author on reasonable request.

## Abbreviations

ABG: Arterial blood gas
ARDS: Acute respiratory distress syndrome
a.u.: Arbitrary units
AVR: Aortic valve replacement
BMI: Body mass index
CABG: Coronary artery bypass graft
COPD: Chronic obstructive pulmonary disease
Crs: Compliance respiratory system
EELI: End-expiratory lung impedance
EIT: Electrical impedance tomography
EtCO2: End-tidal CO2
FCV: Flow-controlled ventilation
FiO2: Fraction of inspired oxygen
GI: Global inhomogeneity index
IBW: Ideal body weight
ICU: Intensive care unit
I:E ratio: Inspiratory:expiratory ratio
IQR: Inter quartile range
MP: Mechanical power
MVP: Mitral valve repair
MVR: Mitral valve replacement
PaCO2: Arterial partial carbon dioxide pressure
PaO2: Arterial partial oxygen pressure
PCV: Pressure-controlled ventilation
Pdrive: Driving pressure
PEEP: Positive end-expiratory pressure
P/F ratio: PaO2/FiO2 ratio
Pmean: Mean airway pressure
Ppeak: Peak pressure
Pplat: Plateau pressure
PV-loop: Pressure–volume loop
ROI: Region of interest
RR: Respiratory rate
RVDi: Regional ventilation delay index
ScvO2: Central venous oxygen saturation
SpO2: Peripheral oxygen saturation
Ti: Inspiratory time
VCV: Volume-controlled ventilation
VILI: Ventilator-induced lung injury
