# Supplementary material for: Flow-controlled ventilation decreases mechanical power in postoperative ICU patients
Source: Intensive Care Med Exp. 2024 Mar 19;12:30. doi: 10.1186/s40635-024-00616-9 (PMC10951187; doi:10.1186/s40635-024-00616-9)
Supplement: Supplementary file 1 — Additional file 1. Supplementary methods and results. [file 40635_2024_616_MOESM1_ESM.docx]

Online Additional

**Flow-controlled ventilation to lower mechanical power in postoperative ICU patients**

Julien P. Van Oosten ^1^, Juliette Francovich^1,2^, Peter Somhorst^1^, Philip van der Zee^1^, Henrik Endeman^1^, Diederik A.M.P.J. Gommers^1^, Annemijn H. Jonkman^1^

**Author affiliations:**

1) Intensive Care, Erasmus Medical Center, Rotterdam, The Netherlands

2) Technical Medicine Program, Delft University of Technology, Delft, The Netherlands

EXTENDED METHODS SECTION

**Detailed enrolment criteria at ICU arrival**

Confirmation of eligibility was reassessed at ICU arrival with the following enrolment criteria: (1) controlled mechanical ventilation via an endotracheal tube, (2) FiO_2_ ≤50%, (3) positive end-expiratory pressure (PEEP) ≤10 cmH_2_O. Exclusion criteria were: (1) severe sputum stasis or production requiring frequent bronchial suctioning (>5 times per nurse shift), (2) severe respiratory insufficiency (PaO_2_/FiO_2_ <100mmHg, or moderate-to-severe ARDS according to the Berlin definition[1]), (3) untreated pneumothorax (i.e., no pleural drainage), (4) hemodynamic instability defined as mean arterial pressure <60mmHg not responding to fluids and/or vasopressors or a noradrenalin dose >0.4mcg/kg/min, (5) contraindications to EIT monitoring (e.g., excessive subcutaneous emphysema, thoracic wounds or bandages limiting belt placement), and (6) an inner tube diameter ≤6mm.

**Continuous monitoring**

Continuous EIT monitoring was initiated with a belt placed at the 4^th^-5^th^ intercostal space (PulmoVista 500, Dräger Medical, Lübeck, Germany). To limit interference with the EIT signals, automated anti-decubitus mattresses were switched off for the duration of the protocol. Subsequently, a conventional tube adapter (CTA) (Ventinova Medical BV, Eindhoven, The Netherlands) and flow sensor (Hamilton Medical, Bonaduz, Switzerland) were placed in between the endotracheal tube and ventilator tubing. The CTA is also an essential part for the FCV mode to operate (Evone ventilator, Ventinova Medical BV, Eindhoven, The Netherlands) and consists of a small intratracheal pressure probe. Output of this pressure probe and of the flow sensor were connected to a dedicated signal acquisition system with pressure amplifiers (MP160, BIOPAC Systems Inc., USA) for continuous and synchronized recording of raw tracings sampled at 200 Hz (AcqKnowledge, BIOPAC Systems Inc., Goleta, USA). Thus, pressure and flow were measured at the exact same location within the circuit throughout the whole study (during both PCV and FCV). Routine clinical monitoring was performed using electrocardiography, peripheral pulse oximetry, arterial blood pressure measurement and central venous pressure measurement (Infinity C700, Dräger Medical, Lübeck, Germany).

**Study procedures**

The study protocol was initiated directly after surgery when the patient arrived on the ICU. No standard recruitment maneuvers were performed after weaning from the cardiopulmonary bypass or before the start of the study protocol. Study steps are presented in Figure 1 of the main manuscript. All measurements were performed with a passive patient (RASS ≤-3) in supine position. Arterial blood gases (ABG), central venous blood gases, SpO_2_, hemodynamic (heart rate, systolic and mean arterial pressures (SBP, MAP) and dose noradrenalin) and a set of respiratory mechanic measurements (end-inspiratory and end-expiratory occlusions for measurements of plateau pressure, total PEEP and resistance) were obtained at the end of each step.

- *Baseline: PCV.* PCV (Evita Infinity V500, Dräger Medical, Lübeck, Germany) settings were first optimized according to local clinical protocol: PEEP as per a decremental PEEP-trial aiming for the highest dynamic compliance (maximum PEEP 24cmH_2_O), FiO_2_ aiming for a SpO_2_ of 95-100% and PaO_2_ <15kPa, peak pressure (Ppeak) resulting in tidal volumes (TV) of 6-8 mL/kg IBW, respiratory rate aiming for a minute ventilation with an end-tidal CO_2_ (EtCO_2_) and PaCO_2_ between 4.5-6.5 kPa, and an I:E-ratio aiming for a brief zero flow phase at the end of inspiration and expiration. These settings were kept for a minimum of 15 minutes to reach a stable condition before initiating continuous EIT, pressure and flow recordings for another 10 minutes at this step.
- *Step 1: Switch to FCV with ‘similar’ PCV settings.*To directly compare FCV and PCV, the ventilation mode was then switched to FCV with the same PEEP and FiO_2_ settings as baseline. Ppeak was titrated to reach the same TV as with PCV. Continuous set flow (determining the minute ventilation) was titrated to maintain a stable EtCO_2_. Inherent to the FCV working mechanism with I:E-ratio of 1:1, respiratory rate is the direct result of the combination between the set flow, the pressure difference between PEEP and Ppeak and the patient’s respiratory mechanics (resistance and compliance). Settings were kept for 30 minutes.
- *Step 2: FCV initial optimization.* To maximally explore the FCV working mechanism, FCV was optimized according to the ABG at the end of step 1 and the manufacturer’s instructions. PEEP was kept constant and FiO_2_ was adapted, if necessary, based on the PaO_2_ and target values mentioned at baseline. Ppeak was titrated in steps of 1 cmH_2_O to reach the highest dynamic compliance: if TV increased more than expected (based on the dynamic compliance) when increasing the Ppeak, then Ppeak was further increased with 1 cmH_2_O. This sequence was repeated until tidal volumes did not increase more than expected (i.e., decreasing dynamic compliance) or until a safety limit of 10mL/kg IBW was reached. Flow was adjusted to maintain PaCO_2_ within target values. Settings were kept for 30 minutes.
- *Step 3: FCV final optimization.*Based on the ABG at the end of step 2, flow and FiO_2_ were adjusted, if necessary, to maintain PaO_2_ and PaCO_2_ within target values. Settings were kept for 30 minutes.

The patient’s management was then resumed as per local clinical protocol (with PCV settings similar as baseline) while data were analyzed offline.

*Flow and pressure tracings*

Signal processing and analyses of flow and intratracheal pressure tracings were done with a custom software (Matlab 2021a, MathWorks, USA). Low-pass filter with cutoff frequency of 20 Hz was applied to remove higher frequency noise.

Breath-by-breath analysis was performed for a period of 8-10 minutes at the end of each step. From the flow tracings, peak inspiratory flow, inspiratory time (Ti), respiratory rate (RR), TV (time-integral of inspiratory flow) and minute ventilation were calculated. Ppeak, total PEEP, and mean pressure (Pmean = ((Ti x RR)/60) x (Ppeak – PEEP) + PEEP)), for both PCV and FCV) were derived from the pressure waveforms.

Pressure-volume (PV) loops were constructed from the volume and pressure tracings (see **Figure S1** for a representative example and parameters). Total energy per breath was computed as the integral of the PV loop times 0.098 (conversion to Joule), including the elastic dynamic and resistive components, but not the elastic static part (unknown PEEP volume). The MP (Joule/min) was calculated by multiplying this total energy per breath by the RR. Dissipated energy was computed as the hysteresis area of the PV loop per breath, and converted to Joule per Liter. For comparison, we also calculated the MP using bedside formulas, using the linear model from Morris et al. [2] for PCV, and the simplified equation from Gattinoni [3] for FCV, since FCV represents a VCV mode during inspiration with its continuous flow.

***
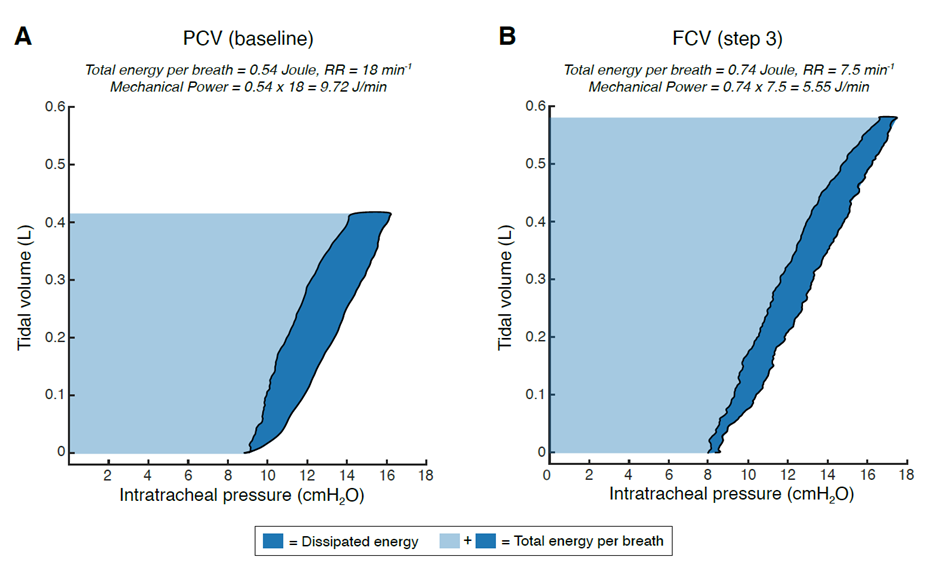
***

***Figure S1.*** *A representative pressure-volume loop obtained in the same patient during pressure-controlled ventilation (PCV) and flow-controlled ventilation (FCV), with corresponding calculations of mechanical power.*

**EIT offline processing and analysis**

*Signal selection*

EIT data were converted using dedicated software (PV500 Data Analysis SW130) and pixel-level data were then processed using a custom software developed in Python. Per patient and using the global impedance signal, a stable period of at least 10 breaths was manually selected at the end of each step (baseline, step 1, step 3). Peak detection algorithm was applied to select the start (nadir) and end (peak) of each inspiration. Then, for the global impedance signal and also for each pixel in the EIT image (totaling 32x32=1024 pixels), an average inspiratory impedance signal was calculated over these 10 breaths. **Figure S2** shows an example of such stable period for the global impedance signal (*left panel*) and the resulting average inspiratory signal of these breaths (*right panel*). For this average breath, signal baseline correction was performed, and inspiratory times were normalized to allow comparisons within and between patients, since respiratory rate varied between the different breaths/steps.

***
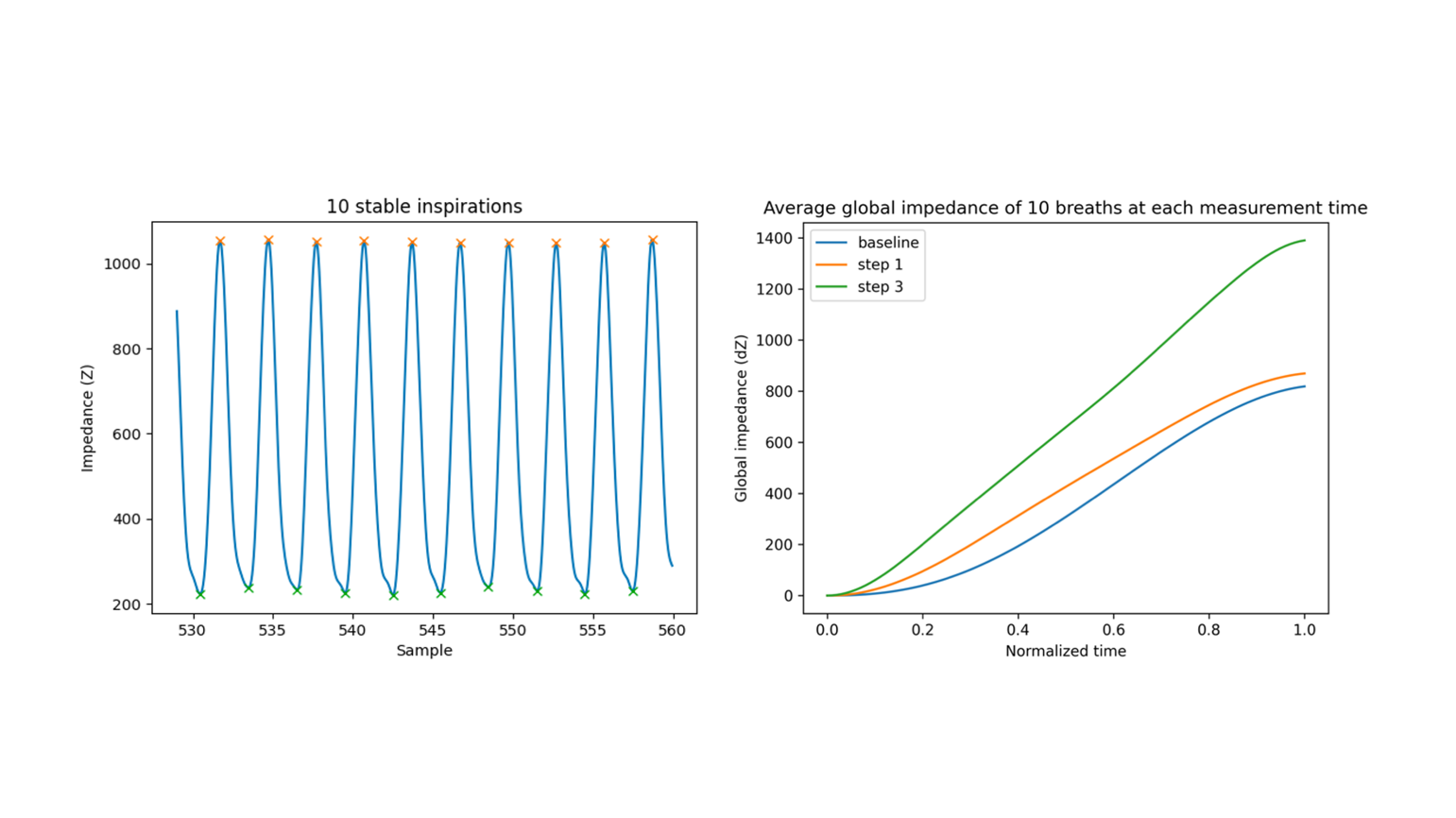
Figure S2.*** *Left: example of 10 stable inspirations at baseline pressure-controlled ventilation (PCV). Right: example of average global impedance over 10 selected inspirations normalized over time at each step (baseline, step 1, step 3). Note that the average global impedance for flow-controlled ventilation (step 1 and step 3) has a more linear shape as compared to baseline PCV, inherent to the working principle of FCV.*

*Determination of ventilated lung space*

No signal filtering was performed to avoid information loss, as this could especially influence temporal ventilation distribution analysis (see below); however, only pixels with a tidal impedance change (∆Z) of at least 15% of maximum pixel ∆Z were included in the further analysis, assuming a significant contribution to the ventilated lung space and to minimize influence of cardiac-related artefacts (**Figure S3**). If applying this threshold resulted in separate clusters of pixels (i.e., remaining artefacts), only the largest cluster of adjacent pixels was assumed to represent the functional lung space and included in the analysis (**Figure S3**). This 15% threshold was chosen in line with [4] and was also visually considered the best cut-off to lower influence of artefacts while minimizing information loss.


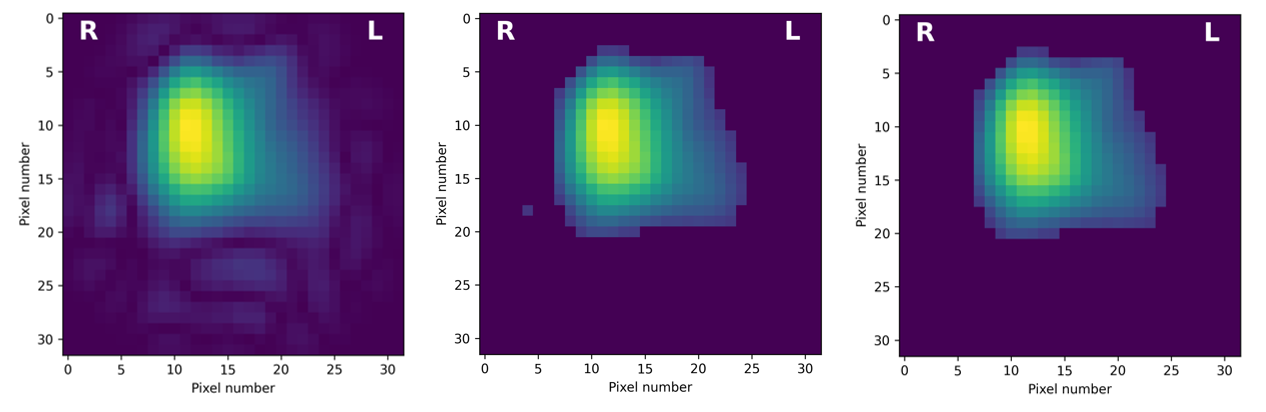


***Figure S3.*** *Left: example of a pixel tidal impedance (∆Z) map at baseline, before determining the functional lung space. Middle: pixels with a ∆Z <15% of maximum pixel ∆Z were excluded. Right: pixel tidal impedance map representing the ventilated lung space, after automatically removing pixels that were not adjacent to the largest cluster. In this example, only 1 pixel (middle figure: row 18, column 4) was additionally removed.*

*Defining regions of interest*

Defining regions of interest (ROI) in a robust way is crucial to analyze subtle changes in regional ventilation distribution among the different ventilator modes. Simply dividing the EIT image into 2 or 4 horizontal slices based on the ventro-dorsal diameter (e.g., 2 ROIs of 16x32 pixels, or 4 ROIs of 8x32 pixels) does not allow for detecting subtle changes in ventilation heterogeneity, since the functional lung space often covers only part of the total EIT field. Furthermore, for a more physiological definition, we wanted to define 4 ROIs (ventral, mid-ventral, mid-dorsal and dorsal) based on the pixels’ contribution to the functional lung space instead of simply dividing the functional lung space into 4 equal-sized regions. Therefore, ROIs were defined using an average pixel impedance map of the three study steps, with each ROI representing to contribute to 25% of total impedance variation for this average map (**Figure S4**). In detail:

1. We first computed an average impedance map of all three study steps (baseline, step 1, and step 3). Hence, all pixels that contributed to ventilation in any step were included in the definition of the ROI.
2. Then, the ROIs were defined with each ROI representing exactly 25% of the total tidal variation in lung impedance (∆Z) of this average pixel impedance map. However, this yields the complexity that a division of 25% often lies somewhere within a pixel row (i.e., it is rare that a full pixel row adds up to exactly 25% of the total ∆Z) (**Figure S5**). Hence, one pixel row could contribute to two ROIs. In such case, we applied a correction on such full row: for instance, if the division between the ventral and mid-ventral region was at 40% of a given row, we added 40% of the ∆Z of this row to the ventral ROI, and the remaining 60% of the ∆Z of this row to the mid-ventral ROI. This approach also prevented differences between the left and right lung to influence the ROI definition.
3. This ROI division was then applied to the original impedance map of each step, to allow within-patient comparisons and quantification of subtle changes in tidal impedance variation for the different regions.


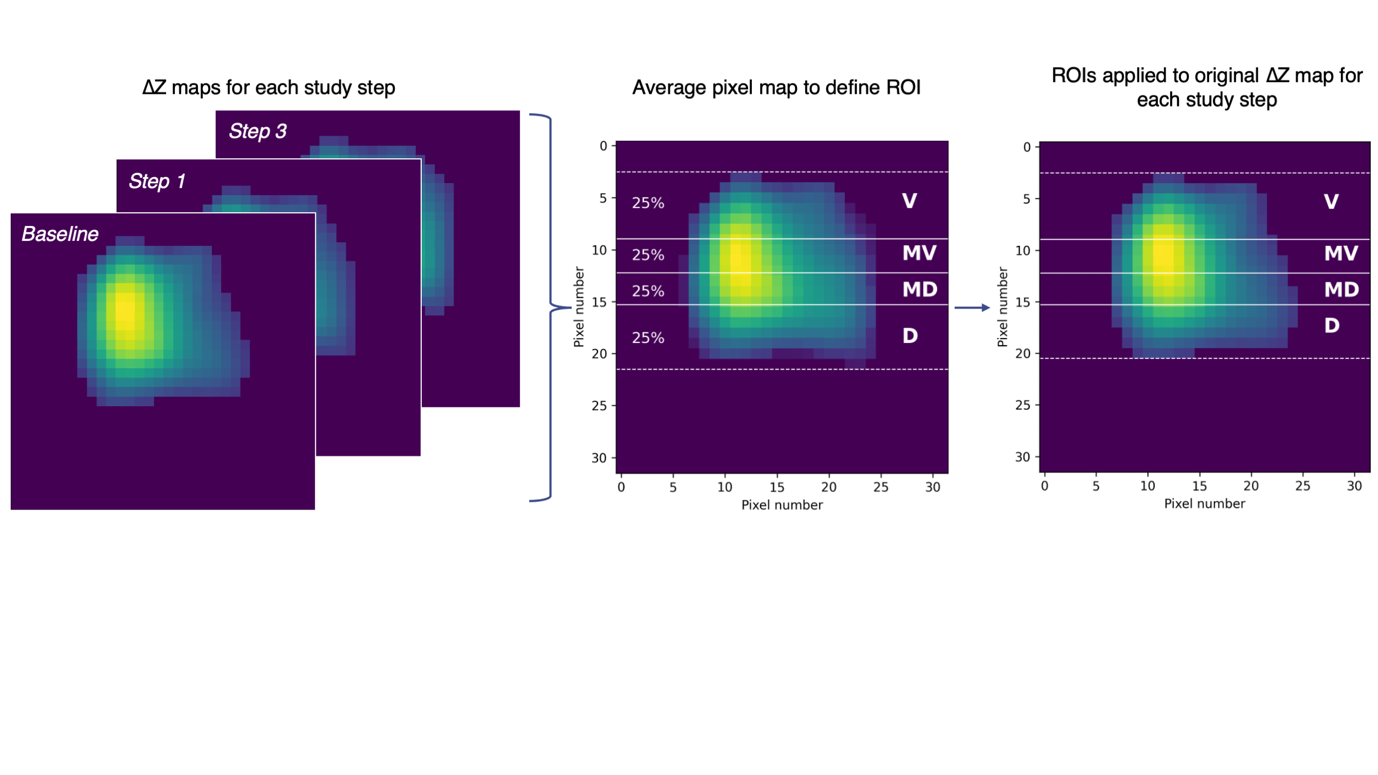


***Figure S4.*** *Example of an average pixel impedance map created with the impedance maps at baseline, step 1 and step 3. The regions of interest (ROI; ventral (V), mid-ventral (MV), mid-dorsal (MD) and dorsal (D)) each represent exactly 25% of the total tidal impedance variation of this average pixel impedance map. Note that the boundary of an ROI could lie within one pixel row (****Figure S5****), which was accounted for (see text for details). This ROI division was then applied to the original impedance maps of each step for further computation of parameters.*


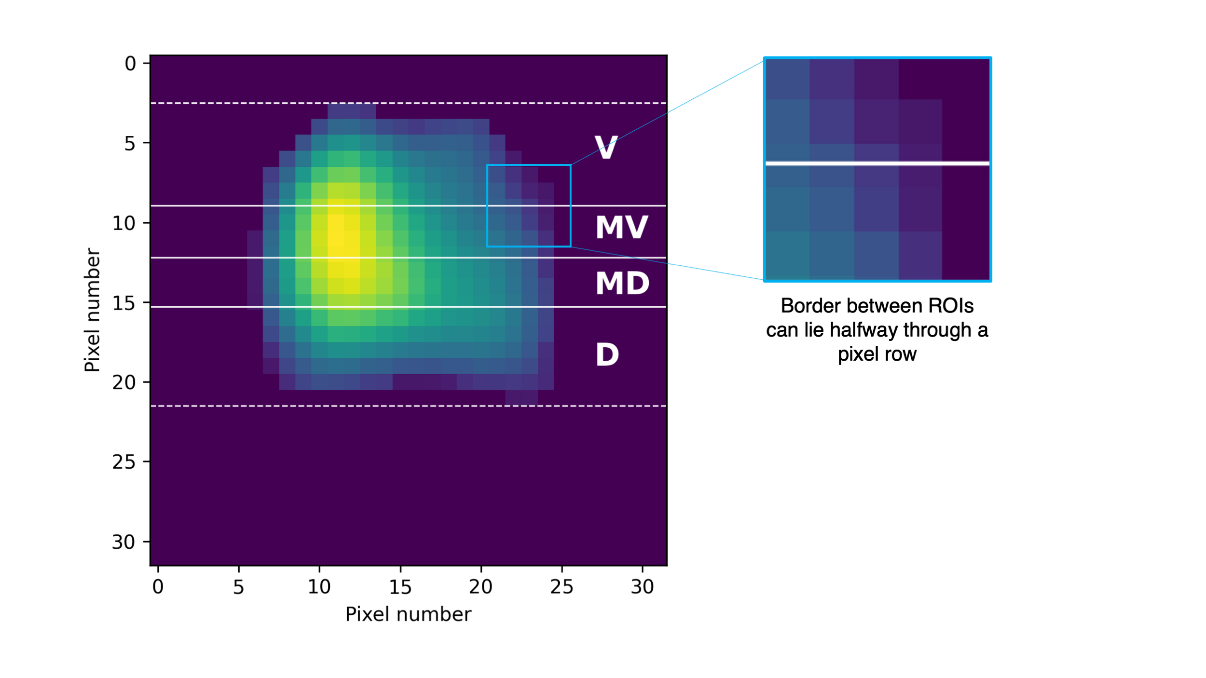


***Figure S5.*** *Example to illustrate that ROI division (ventral (V), mid-ventral (MV), mid-dorsal (MD) and dorsal (D)) can lie in between a pixel row. Dotted lines reflect the boundary of the functional lung space (i.e., ventilated pixels).*

*Computation of EIT parameters*

The following parameters were computed for each study step, using the average breath from each stable period (as in **Figure S2**):

- ***Tidal impedance variation (∆Z):*** the global ∆Z and regional ∆Z (per ROI) were calculated as the amplitude of the respective global and regional impedance signal during inspiration (peak minus nadir).
- ***Static compliance:*** the global static compliance and regional static compliance (per ROI) were calculated as the ratio of ∆Z divided by the driving pressure, with driving pressure measured as plateau pressure minus total PEEP derived from the intratracheal pressure tracings during both PCV and FCV.
- ***Global end-expiratory lung volume (EELI):*** computed as the baseline of the global impedance signal.

Furthermore, we computed the following parameters to visualize and quantify the overall, spatial, and temporal homogeneity of lung ventilation:

- ***Global inhomogeneity index (GI):*** as a measure for overall homogeneity of ventilation and as per [5]: GI(%)=((∑[x,y∈_lung_]|∆Z_xy_–Median(∆Z_lung_)|)/(∑[x,∈_lung_]∆Z_xy_))x100; ∆Z_xy_ represents the impedance change of a ventilated pixel (x,y), and ∆Z_lung_ the impedance change of the total ventilated lung area. A lower GI thus reflects a more homogeneous ventilation distribution.
- ***Spatial homogeneity:*** spatial homogeneity was evaluated in two ways:
  - First, to provide a visualisation of the *continuous* inspiratory volume distribution over all ROIs, the impedance waveforms per ROI were normalized over time and visualized as a percentage of the global ∆Z (see **Figure S6**).

| 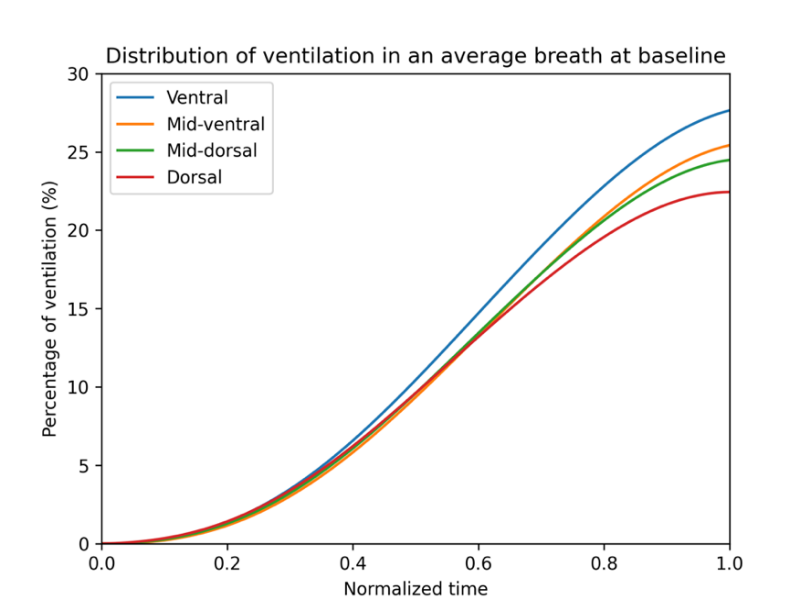 | ***Figure S6.*** *Example of a continuous regional volume distribution (per region of interest) in an average inspiration at baseline PCV.* |
| --- | --- |

- Second, the regional intra-tidal impedance distribution was visualized by dividing the global inspiration into five parts of equal ∆Z and plotting the impedance changes for each ROI (see **Figure S7**), in line with Lowhagen et al.[6]

| 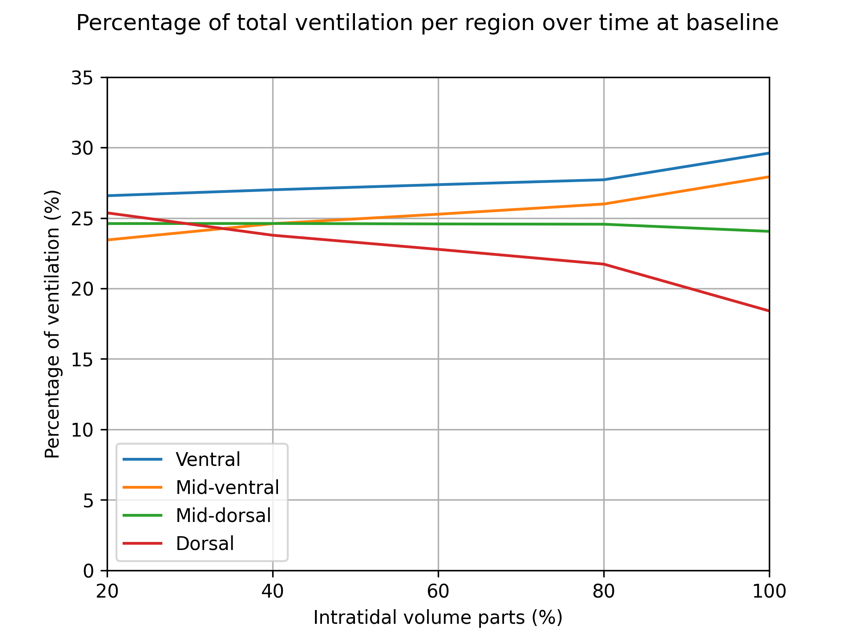 | ***Figure S7.*** *Example of regional intra-tidal volume distribution per region of interest in an average inspiration at baseline PCV. The inspiration was divided into five equal parts of ∆Z (intra-tidal volume parts, each representing 20% of total ∆Z). Note that throughout the inspiratory phase, ventilation is distributed more to the ventral regions in this example.* |
| --- | --- |

- ***Temporal homogeneity:*** The regional ventilation delay index (RVDi) was used as a measure of temporal homogeneity of lung inflation.[7] Regional ventilation delay (RVD) was first computed for each pixel of the ventilated lung space as RVD = Δt_RVD_/Δt_max–min_, with Δt_RVD_ the time between start of inspiration (as per the global ∆Z) until pixel ∆Z reached 40% of the maximal ∆Z, normalized to global inspiration time (Δt_max–min_) and expressed as percentage (see **Figure S8**). RVDi was then calculated as the standard deviation of all pixel RVDs. A lower RVDi thus reflects a more homogeneous lung inflation.

| *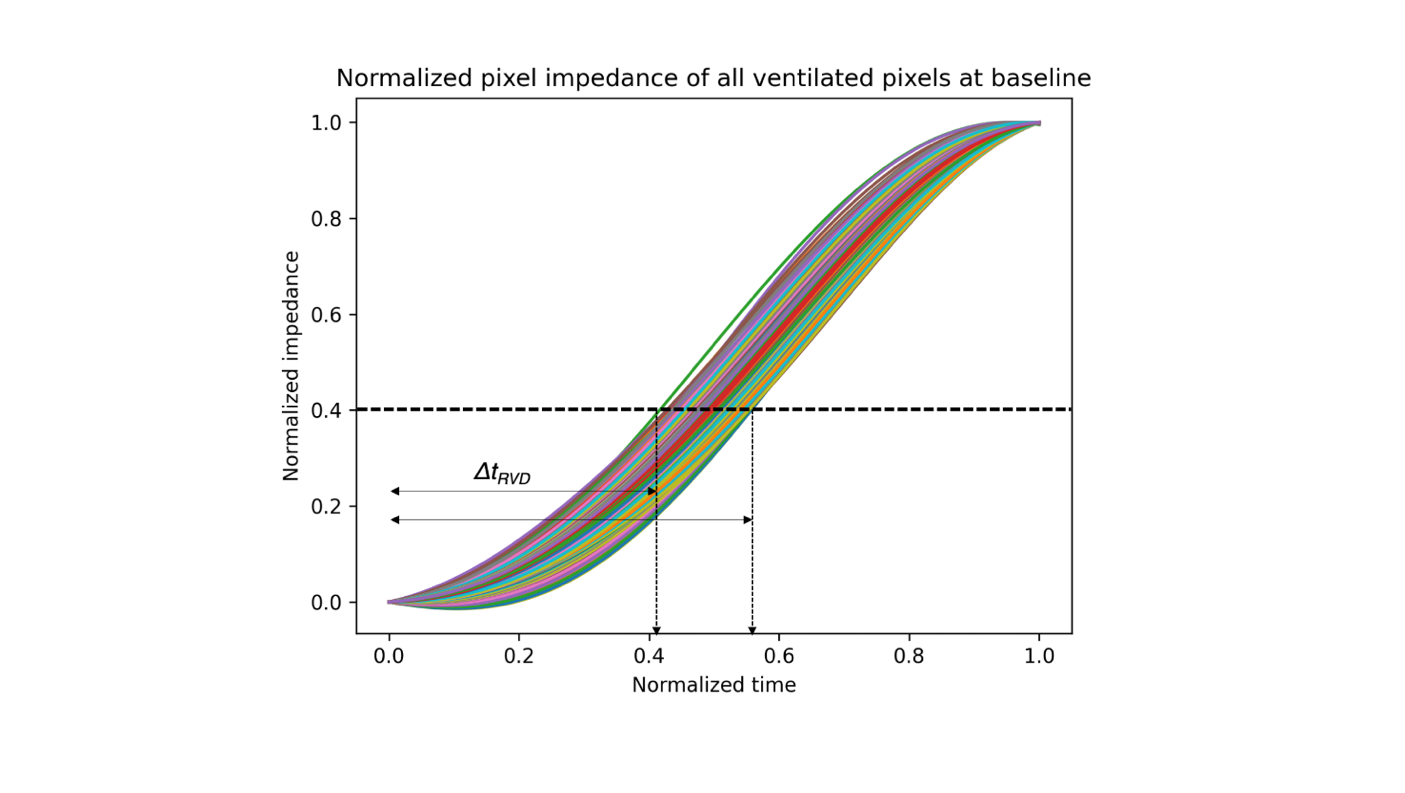* | ***Figure S8.*** *Example of the tidal impedance of all separate pixels involved in the ventilated lung space at baseline PCV, normalized for time and impedance. Δt_RVD_ is the time between start of inspiration (as per the global ∆Z) until pixel ∆Z reached 40% of the maximal ∆Z (dotted horizontal line at 0.4). The dotted vertical lines indicate the first and last pixel that reach the threshold of 0.4 (smallest and largest Δt_RVD_). The RVD inhomogeneity (RVDi) is computed as the standard deviation of the Δt_RVD_ of all pixels in the ventilated lung space.* |
| --- | --- |

**Sample size**

No studies exist assessing either lung aeration or mechanical power during both PCV and FCV in an adequate manner. Therefore, our sample size was based on the reported difference in minute volume between conventional mechanical ventilation (CMV) and FCV in healthy pig lungs: 5.5±0.2L/min for FCV vs. 7.0±1.0 L/min for CMV [8]. Using G*Power (Statistical Power Analyses, Universität Düsseldorf, Germany) and a matched pairs T-test approach with alpha of 0.05 and a power of 0.80, the required sample size was 6 patients. To compensate for the fact that the effect size of FCV on other parameters is unknown, we planned to enroll a total of 10 patients for the full protocol.

EXTENDED RESULTS SECTION

**Population**

One non-serious adverse event was reported and included a patient-ventilator dyssynchrony when removing the EIT belt after study completion, which required extra sedation and muscle relaxant.

**Additional results of FCV in patients with high airway resistance**

Two patients demonstrated a relatively high respiratory system static compliance (49.2 and 57.4 ml/cmH_2_O) in combination with a high airway resistance (15.5 and 7.4 cmH_2_O/L/s), of which one patient was previously diagnosed with COPD. Also, in these patients, ventilation with FCV resulted in a lower net driving pressure and reduction in the airway resistance, MP, dissipated energy and minute volume compared to PCV, despite that tidal volumes were slightly increased to optimize FCV (see **Table S3**).

**Table S1.** Results PCV (baseline) vs. FCV with PCV settings (step 1)

| Parameter | **PCV baseline**  **Median (IQR)** | **FCV step 1**  **Median (IQR)** | **P-value** |
| --- | --- | --- | --- |
| ***Respiratory parameters*** | | | |
| TV/IBW inspiratory (mL) | 6.0 (5.5-7.1) | 6.3 (5.5-7.1) | 1.000 |
| Driving pressure (cmH_2_O) | 9.2 (7.7-11.7) | 9.6 (8.0-12.5) | 1.000 |
| PEEP set (cmH_2_O) | 7.5 (6.4-8.0) | 8.0 (6.8-8.0) | 1.000 |
| PEEP total (cmH_2_O) | 8.3 (7.5-9.2) | 8.8 (8.1-9.6) | 0.027 |
| Ppeak set (cmH_2_O) | 20.0 (18.8-22.0) | 19.0 (18.0-20.5) | 1.000 |
| Ppeak (cmH_2_O) measured | 18.6 (16.8-21.5) | 19.8 (17.4-21.8) | 0.669 |
| Pplat (cmH_2_O) | 17.5 (16.2-20.5) | 18.6 (17.3-21.0) | 0.534 |
| Pmean (cmH_2_O) | 12.6 (11.0-13.4) | 13.6 (12.5-14.7) | 0.031 |
| Crs stat (mL/cmH_2_O) | 44.5 (36.1-52.7) | 41.9 (35.7-52.8) | 1.000 |
| Resistance (cmH_2_O/l/s) | 13.8 (12.4-14.9) | 7.9 (7.4-8.9) | 0.004 |
| RR (x/min) | 18 (17.5-20.0) | 15.6 (14.3-18.7) | 0.221 |
| Minute Volume (L/min) | 8.0 (6.5-8.4) | 6.7 (6.0-7.5) | 0.438 |
| Mechanical Power (J/min) | 11.0 (8.5-12.8) | 9.4 (8.0-11.1) | 0.286 |
| Mechanical Power, bedside formulas (J/min) | 13.2 (10.5-15.2) | 9.7 (8.4-11.0) | 0.057 |
| Dissipated Energy (J/L) | 0.34 (0.21-0.43) | 0.22 (0.17-0.26) | 0.008 |
|  |  |  |  |
| ***Gas exchange parameters*** | | | |
| P/F ratio | 324 (241-365) | 316 (255-363) | 1.000 |
| PaO_2_ (kPa) | 14.3 (12.9-17.7) | 14.2 (13.4-14.9) | 0.987 |
| PaCO_2_ (kPa) | 5.4 (5.1-6.2) | 5.3 (5.0-5.9) | 1.000 |
| Ventilatory ratio | 1.20 (1.11-1.31) | 1.07 (0.92-1.30) | 0.791 |
|  |  |  |  |
| ***Hemodynamic parameters*** | | | |
| Arterial-venous delta CO_2_ (kPa) | 1.06 (0.86-1.13) | 0.83 (0.61-1.09) | 1.000 |
| ScvO_2_ (%) | 71.2 (64.7-75.8) | 69.0 (65.6-76.6) | 1.000 |
| Dose noradrenalin (ug/kg/min) | 0.11 (0.05-0.16) | 0.10 (0.04-0.15) | 0.456 |

***Abbreviations:*** *Crs: Compliance respiratory system; FCV: Flow-Controlled Ventilation; IBW: Ideal Body Weight; IQR: Inter Quartile Range; PCV: Pressure-Controlled Ventilation; Ppeak: Peak pressure; PEEP: Positive End-Expiratory Pressure; Pmean: mean airway pressure; Pplat: Plateau pressure; PaO_2_: arterial partial oxygen pressure; PaCO_2_: arterial partial carbon dioxide pressure; P/F ratio: PaO_2_/FiO_2_ ratio; RR: Respiratory Rate; ScvO_2_: central venous oxygen saturation; TV: Tidal Volume*

**Table S2.** EIT results PCV (baseline) vs FCV with PCV settings (step 1); values represent median (IQR)

| **Table S2a. Changes in EIT parameters during FCV as compared to PCV^*^** | | | |
| --- | --- | --- | --- |
|  | | **FCV step 1** | **P-value** |
| Global change in ΔZ (%) | | 5.0(-3.4-11.2) |  |
| Regional change in ΔZ (%)  ROI ventral  ROI mid-ventral  ROI mid-dorsal  ROI dorsal | | -3.6(-8.5-4.6)  3.7(-7.1-7.1)  7.0(-4.6-10.1)  20.8(-1.0-24.4) | 0.091^1^ |
| Global change in static compliance (%) | | -4.0 (-9.0-4.9) |  |
| Regional change in static compliance (%)  ROI ventral  ROI mid-ventral  ROI mid-dorsal  ROI dorsal | | -12.2 (-14.8-6.7)  -6.0 (-10.7-3.9)  -2.8 (-8.7-1.9)  6.4 (0.2-9.8) | 0.050^2^ |
| Change in global EELI (a.u.) | | 29 (-38-64) | 1.000 |
| **Table S2b. Absolute EIT parameters reflecting lung and ventilation homogeneity** | | | |
|  | **PCV** | **FCV step 1** | **P-value** |
| GI (%) | 43.8 (41.4-45.3) | 43.8 (40.6-45.9) | 1.000 |
| RVDi (%) | 2.75 (2.28-4.63) | 3.94 (3.60-5.80) | 0.264 |

***Abbreviations:*** *a.u.: arbitrary units; EELI: End-Expiratory Lung Impedance; EIT: Electrical Impedance Tomography; FCV: Flow-Controlled Ventilation; GI: Global Inhomogeneity index; PCV: Pressure-Controlled Ventilation; ROI: Region Of Interest; RVDi: Regional Ventilation Delay index.*

*^*^Changes in ΔZ and static compliance are expressed as percentage change between FCV step 1 and PCV at baseline, since both are expressed in arbitrary units which makes direct comparisons between patients unreliable.*

*^1^p-value reflects the non-significant difference between PCV baseline vs. FCV step 1 regarding the distribution of ΔZ among the four ROIs, using a Kruskall Wallis test on the percentage changes from baseline (to account for the fact that ΔZ is measured in arbitrary units). ^2^p-value reflects the non-significant difference between PCV baseline vs. FCV step 1 regarding the distribution of the change in static compliance among the four ROIs, using a Kruskall Wallis test on the percentage changes from baseline (to account for the fact that ΔZ and thereby also the static compliance is measured in arbitrary units).*

**Table S3.** Results PCV (baseline) vs optimized FCV (step 3) in two patients with high airway resistance and high respiratory system compliance.

|  | **PCV baseline**  **Mean** | **FCV step 3**  **Mean** |
| --- | --- | --- |
| ***Respiratory parameters*** | | |
| TV inspiratory (mL) | 464 | 498 |
| Driving pressure (cmH_2_O) | 10.1 | 8.7 |
| PEEP total (cmH_2_O) | 8.4 | 9.5 |
| Ppeak set (cmH_2_O) | 24.5 | 18.5 |
| Ppeak measured (cmH_2_O) | 19.7 | 19.3 |
| Pplat (cmH_2_O) | 18.5 | 18.2 |
| Crs stat (mL/cmH_2_O) | 49.2 | 57.4 |
| Resistance (cmH_2_O/l/s) | 15.5 | 7.4 |
| RR (x/min) | 21.0 | 12.5 |
| Minute Volume (L/min) | 9.8 | 5.8 |
| Mechanical Power (J/min) | 15.7 | 8.5 |
| Dissipated Energy (J/L) | 0.36 | 0.22 |
|  |  |  |
| ***Gas exchange parameters*** | | |
| PaCO_2_ (kPa) | 5.9 | 5.8 |
| Ventilatory ratio | 2.0 | 1.2 |

***Abbreviations:*** *Crs: Compliance respiratory system; FCV: Flow-Controlled Ventilation; PCV: Pressure-Controlled Ventilation; Ppeak: peak pressure; PEEP: Positive End-Expiratory Pressure; Pplat: plateau pressure; PaCO_2_: arterial partial carbon dioxide pressure; RR: Respiratory Rate; TV: Tidal Volume*

**Influence of patient management on EELI reliability**

**
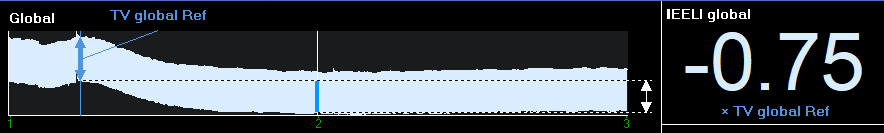
**

***Figure S9.*** *Example of the influence of a fluid bolus on the end-expiratory lung impedance. Patient received 850ml of cellsaver blood in a fast manor postoperatively which decreased the EELI value with 0.75 points without any change in PEEP, tidal volume or gas exchange. Therefore, the EELI as a parameter of lung aeration was inappropriate in our study population.*

*
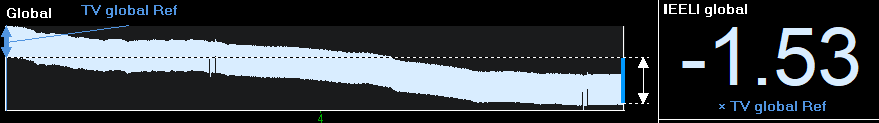
*

***Figure S10.*** *Another example of the influence of a fluid bolus on the end-expiratory lung impedance. Patient slowly received 700ml of crystalloid fluids postoperatively for hypotension which decreased the EELI value with 1.53 points without any change in PEEP, tidal volume or gas exchange.*

**Additional EIT results: Inspiratory regional impedance distribution**

Average continuous inspiratory regional impedance distribution over all patients showed a trend towards more participation of the dorsal lung region towards the end of inspiration in step 1 and step 3 as compared to baseline PCV, with significantly more dorsal participation with optimized FCV (step 3). **Figure S11** is an extension of Figure 3 of the main manuscript, with study step 1 added for better visual comparison. Similar results can be observed when visualized using the regional intra-tidal impedance distribution, averaged per ROI for all patients (see **Figure S12**).

**
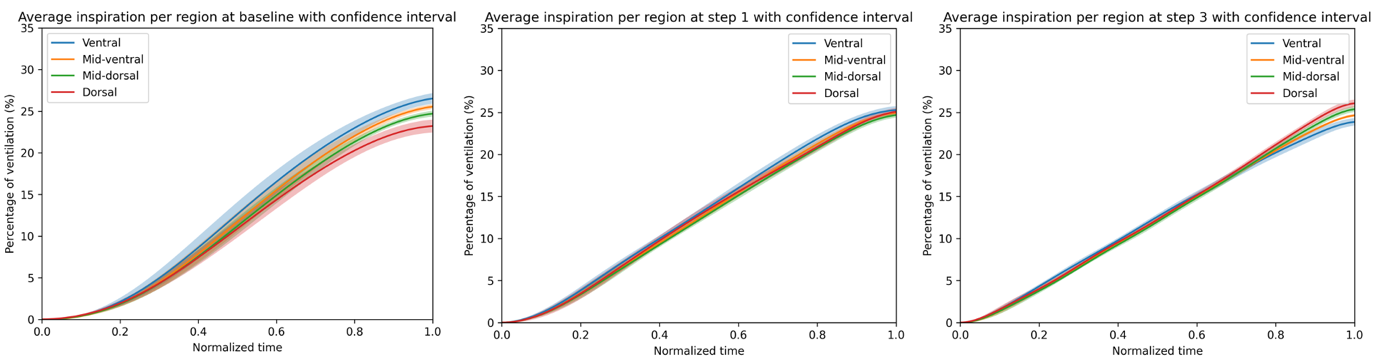
**

***Figure S11.*** *Continuous regional impedance distribution averaged over all patients and per region of interest, in an average inspiration at baseline, step 1, and step 3 (left to right). Shaded areas represent 95% confidence intervals.*

**
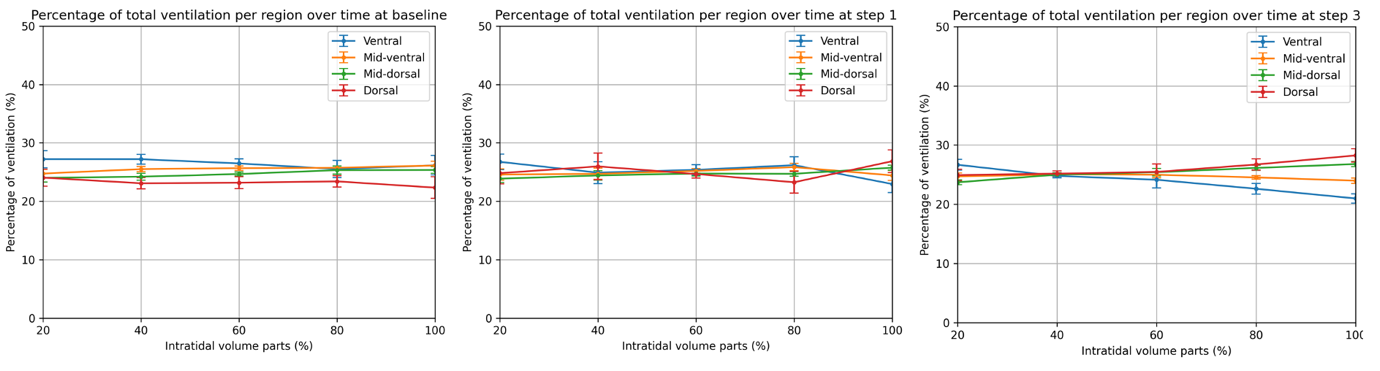
 *Figure S12.*** *Regional intra-tidal impedance distribution averaged over all patients per region of interest in an average inspiration at baseline, step 1, and step 3 (left to right). The inspiration was divided into five equal parts of ∆Z. Error bars represent 95% confidence intervals.*

REFERENCES

1. The ARDS Definition Task Force. **Acute Respiratory Distress Syndrom – The Berlin Definition.***JAMA*2012;307(23):2526-2533.
2. Trinkle CA, Broaddus RN, Sturgill JL, Waters CM, Morris PE. **Simple, accurate calculation of mechanical power in pressure controlled ventilation (PCV).***Intensive Care Med Exp*2022;10:22.
3. Giosa L, Busana M, Pasticci I, Bonifazi M, Macri MM, Romitti F, *et al*. **Mechanical power at a glance: a simple surrogate for volume-controlled ventilation.***Intensive Care Med Exp*2019;7:61.
4. Heines SJH, de Jongh SAM, Strauch U, van der Horst ICC, van de Poll MCG, Bergmans DCJJ. **The global inhomogeneity index assessed by electrical impedance tomography overestimates PEEP requirement in patients with ARDS: an observational study.** *BMC Anesthesiol* 2022;22(1):258.
5. Zhao Z, Möller K, Steinmann D, Frerichs I, Guttmann J. **Evaluation of an electrical impedance tomography-based global inhomogeneity index for pulmonary ventilation distribution**. *Intensive Care Med* 2009;35(11):1900-1906.
6. Lowhagen K, Lundin S, Stenqvist O. **Regional intratidal gas distribution in acute lung injury and acute respiratory distress syndrome assessed by electric impedance tomography**. *Minerva Anestesiol* 2010; 76(12):1024-1035.
7. Muders T, Hentze B, Simon P, Girrbach F, Doebler MRG, Leonhardt S, *et al.* **A Modified Method to Assess Tidal Recruitment by Electrical Impedance Tomography.** *J Clin Med* 2019;8(8):1161.
8. Schmidt J, Wenzel C, Mahn M, Spassov S, Schmitz CH, Borgmann S, *et* al. **Improved lung recruitment and oxygenation during mandatory ventilation with a new expiratory ventilation assistance device: A controlled interventional trial in healthy pigs.** *Eur J Anaesthesiol* 2018;35(10):736-744.
